# The use of RP-HPLC–Q-TOF–MS as a powerful tool for wastewater composition profiling and selection of water pollution marker specific to wastewater contamination

**DOI:** 10.1007/s00706-018-2259-y

**Published:** 2018-08-09

**Authors:** Dagmara Kempińska, Agata Kot-Wasik

**Affiliations:** Department of Analytical Chemistry, Faculty of Chemistry, Gdańsk University of Technology, Gdańsk, Poland

**Keywords:** Wastewater analysis, Water pollution profiling, Emerging contaminants, Extraction, High performance liquid chromatography, Mass spectrometry

## Abstract

**Abstract:**

Limited drinking water resources and water pollution are one of the main worldwide problems. To reduce the consumption of fresh water resources, the use of treated wastewater has been proposed. The farmlands have been irrigated with wastewater for centuries, but the composition of used sewage has changed over the years. Recent research has revealed the presence of hundreds of new organic contaminants in many environmental waters, including wastewaters and their receivers. For this reason, wastewater profiling and monitoring are of high importance and urgent need. In this study, the HPLC–Q-TOF–MS has been used for the profiling of wastewater composition and evaluation of the water pollution markers belonging to emerging contaminants. Three different solid-phase extraction approaches were applied to obtain the best results. Compounds such as acesulfame-K, caffeine, carbamazepine, cyclamate, ibuprofen, methyl paraben, paracetamol, or saccharin were detected in raw wastewater samples, whereas only acesulfame-K, carbamazepine, and sucralose were found in effluent samples. It seems that these particular compounds might be chosen as water pollution marker specific to Polish communal sewages.

**Graphical abstract:**

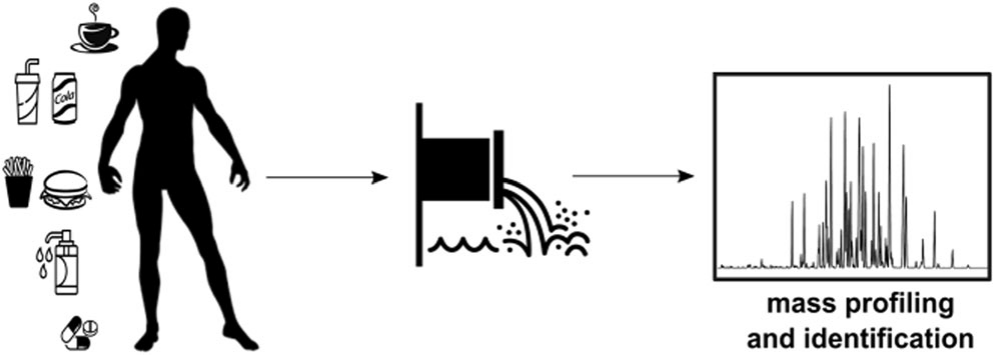

## Introduction

Human activities that are based on water usage must generate wastewater. The United Nations Educational, Scientific and Cultural Organization (UNESCO) evaluates that the amount of sewage produced yearly is almost 1500 km^3^ and it is about six times more than amount of water of rivers that run across all the world. As the general demand for water grows systematically, the quantity of sewage produced and its overall pollution load is continuously changing worldwide. It is estimated that over 80% of the world’s wastewater, and even over 95% in less developed countries, is discharged to the environment without the proper treatment [1]. Once released into water, wastewater is diluted and transported downstream and may infiltrate into groundwater, an important source of drinking water. Finally, river water containing discharged sewage contaminated with pollutants enters seas and oceans, where it may have negative impact on the marine environment [1, 2]. Currently, wastewaters are no longer seen as an environmental problem, but rather as the part of challenging solutions that society faces today. Regarding ever-growing water demand, the sewage has gained a momentum as an alternative source of water supply (not as a source of contamination), changing the idea of wastewater management from ‘treatment and discharge’ to ‘recycle, reuse, and recovery of resources’. Wastewater can be also a cost-effective and sustainable source of organic matter, nutrients or energy. According to the *United Nations World Water Assessment Programme Report 2017: Wastewater. The Untapped Resource* published by UNESCO [1], the prospective advantages of acquiring these resources from sewage go well beyond human health and environmental condition and may impact on energy or food security and climate change mitigation, as well. The introduction of plan of using the partially treated wastewaters and effluents for ecosystem services may reduce freshwater abstractions and allows fisheries as well as other aquatic ecosystems to thrive by recharging depleted aquifers and minimalizing water pollution [1]. However, advance quality control and improvement of wastewater treatment systems are required. Although the use of untreated or diluted wastewater for irrigation has taken place for centuries, their composition has changed over the years. Therefore, their profiling and monitoring are of high importance and need.

So far, water and wastewater quality investigations have been focused on the determination of nutrients, heavy metals, priority compounds such as persistent organic pollutants (POPs) or measurement of physicochemical parameters (e.g., conductivity, temperature or pH). However, last research has revealed the presence of hundreds of organic compounds in wastewater and environmental waters that had not been detected before [3]. These compounds are known as emerging contaminants and are classified as naturally or synthetic occurring chemicals with the potential of entering to natural environment and causing essentially unknown adverse ecological and human health effects. Their presence in waters is not regulated; moreover, they are not even commonly monitored because their determination requires high sensitivity offered by hyphenated techniques [2], such as high performance liquid chromatography coupled with mass spectrometry [4–7] or gas chromatography coupled with mass spectrometry [8–10]. Additionally, their environmental fate and ecotoxicological effects are generally unknown or not fully understood [2]. Nevertheless, due to their good solubility in water, stability in aqueous environment, sustained release, resistance to self-purification processes, and relatively low degradation efficiency during conventional water and wastewater treatment processes, some novel contaminants, such as alkaloids, artificial sweeteners, illicit drugs, pharmaceutical residues, personal care products, or X-ray contrast media, have been proposed to be used as water pollution markers specific to wastewater contamination [4].

The purpose of the work described in this paper is the evaluation of analytical approach that can be applied for the profiling of wastewater composition and determination of the emerging contaminants in wastewater. Three different analytical protocols dedicated to acidic compounds, neutral compounds, and basic compounds have been evaluated and applied for wastewater samples. The potential of reversed-phase high-performance liquid chromatography coupled with quadrupole-time-of-flight mass spectrometry (RP-HPLC–Q-TOF–MS) for the identification of emerging contaminants residue in sewage samples has been established. Finally, pollution markers specific to domestic wastewater in Northern Poland have been proposed.

## Results and discussion

For wastewater profiling, the samples have been analyzed using HPLC–Q-TOF–MS working in SCAN mode (ESI (+) and ESI (−)). Chromatographic performance was studied in terms of resolution, peak shape, and ion suppression. Two main organic solvents were applied for the separation of compounds: methanol and acetonitrile, both mixed with water containing 0.1% FA. Complete separation of the analytes of interest from the matrix compounds that has been achieved with methanol enhanced both: degrees of certainty during identification and elimination of matrix effects during ionization to avoid mutual interactions and competitions.

**Fig. 1:**
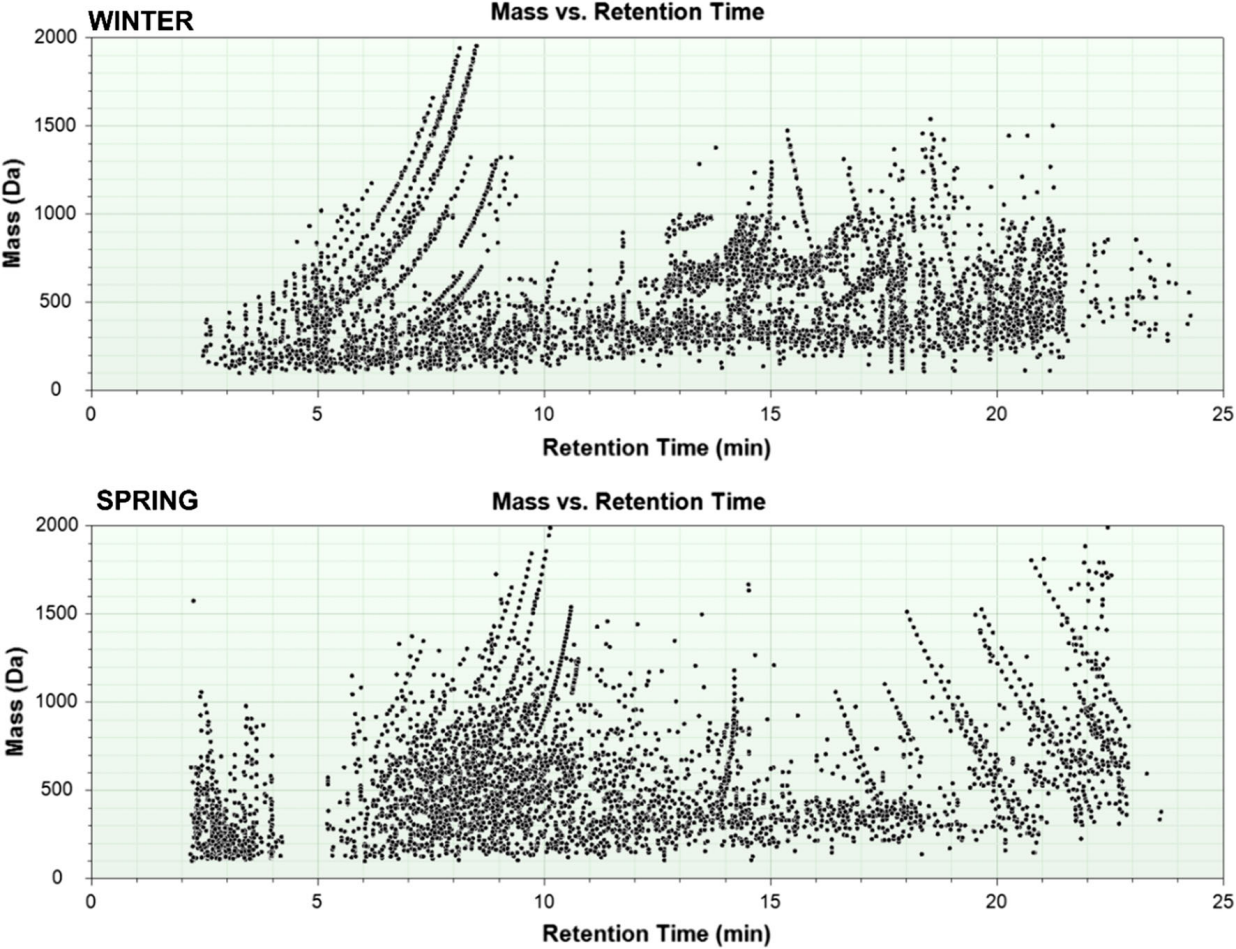
The relationship between the mass of chemical species detected in raw wastewater and the retention time

Each obtained chromatogram LC-HRMS (SCAN mode) has been processed with molecular feature extraction (MFE) mode, with noise threshold set at 1300 units and database search. Identification of unknown compounds by mass spectrometry was possible due to the availability of a correct elemental composition or molecular formula. Because accurate mass measurements alone are often not enough to conclusively determine the formula of unknown compounds, the identification was supported by isotope patterns. During calculations of the total number of possible formulas for a particular ion, a highly effective approach based on the filtering of formulas based on a set of “Seven Golden Rules” [11] has been applied. Moreover, the formula for compounds with a match in a database was treated as correct as long as the mass measurements satisfied particular additional criteria: maximum 2 ppm mass accuracy and 5% absolute isotope ratio deviation. Although allowable mass error window Δppm up to ± 7.5 ppm is quite often taken into account, in case of water and wastewater samples results with a mass error that is greater than 2 ppm were already eliminated from further identification. In case of a list of candidate, molecular formulas that were taken for further identification approve were only with scores > 98%. This significantly reduced false-positive results. The results achieved for raw wastewater sample (both ESI modes) are shown in Fig. 1.

After data processing, list of more than 4000 chemical compounds was obtained. Based on that, significant differences between the composition of raw sewage collected during different seasons could be observed (Fig. 1). In case of raw sewage sample collected in February, the occurrence of two numerous groups of chemical species, such as medium-polar (5–8 min) and non-polar (14–20 min) ones, can be observed, whereas during the analysis of raw wastewater sample collected in May the majority of compounds eluted between 6 and 14 min. This shows the relatively non-polar nature of these chemicals. These differences in composition may result from the lifestyle of local residents and the work of local companies that depend on time of the year. The main factor that affects the sewage composition is tourism, which is one of the main sources of local income. The tourist season begins in May and since then, these areas are visited by millions of people both from Poland and abroad. For this reason, most of the gastronomic locals and other attractions are opened only during this period. Second factor that should be taken into account is fish protection periods. Hence, cutters staying in the port influence the work of the repair ship yard or even local fish factory.

**Fig. 2:**
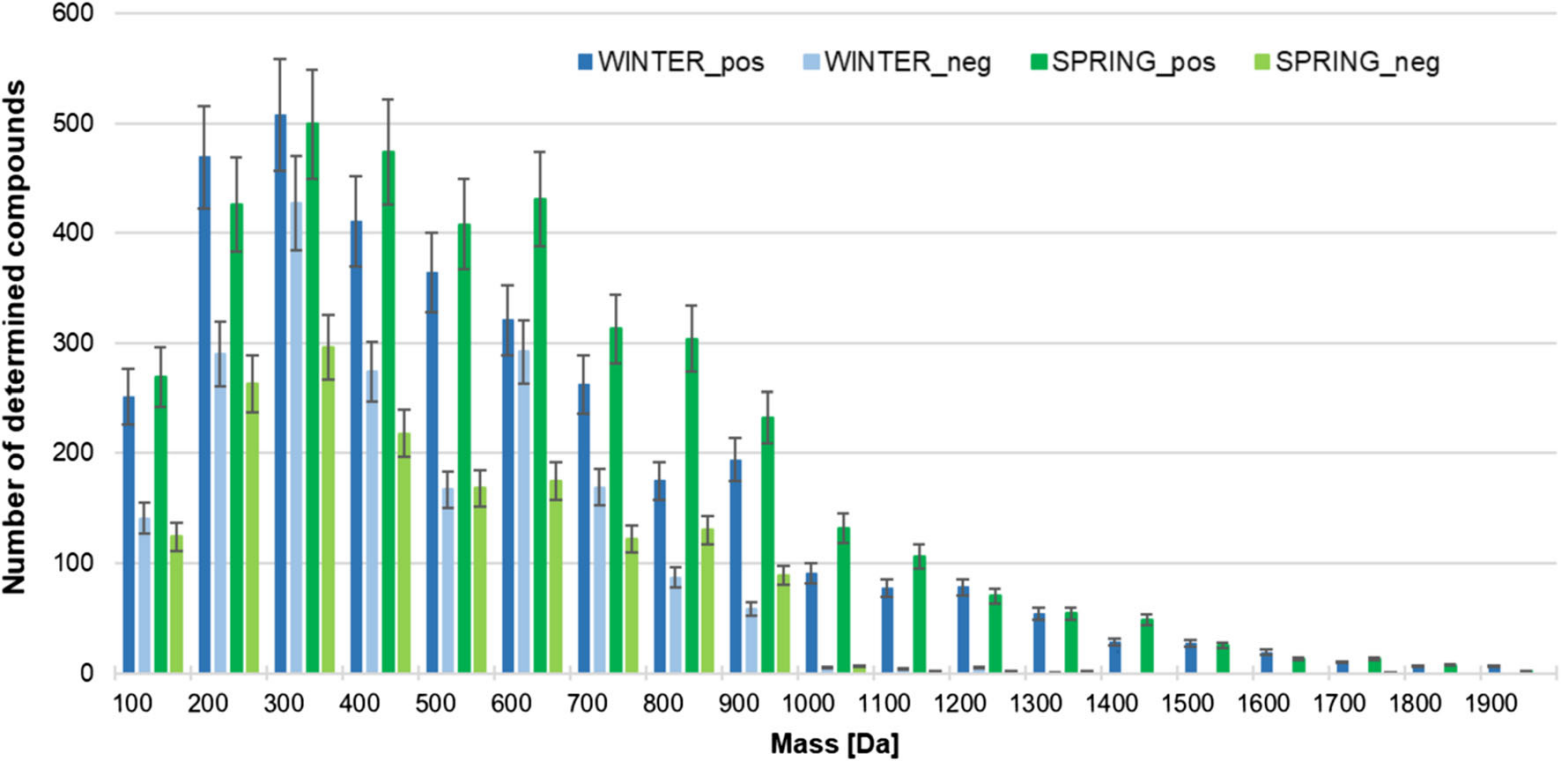
The relationship between number of determined compounds in raw wastewater and their mass

The relationship between the number of detected chemical species in raw sewage and their mass is shown in Fig. 2. It can be observed that the most of compounds determined in raw wastewater samples were identified in the positive ionization mode. It is known that the basic compounds are easily ionized in this mode, so compounds containing amino or amide groups in their structure should be expected. By the contrast, compounds responded in negative ionization mode have functional groups such as carboxy, ester, or hydroxyl group. The presence of individuals with a very diverse chemical structure containing various functional groups cannot be excluded. These compounds ionize in both ion-forming modes. Low-molecular weight compounds (from 100 to 500 Da) were generally determined in raw wastewater sample collected in winter and analyzed in the positive ion formation mode, whereas in case of sample collected in spring, low molecular weight and medium molecular weight compounds (500–1000 Da) were generally detected. In contrary to that during analysis of sewage samples undertaken in negative ionization mode, low and medium molecular weight compounds were generally determined in case of samples collected in winter time. A relatively small number of compounds with mass above 1000 Da were found in the samples in both ionization modes. This does not mean that they do not occur in wastewater. The extraction approaches developed as a part of this study were focused on the extraction of low molecular weight compounds, so it is possible that the use of other conditions would result in greater recovery of these chemical individuals.

**Fig. 3:**
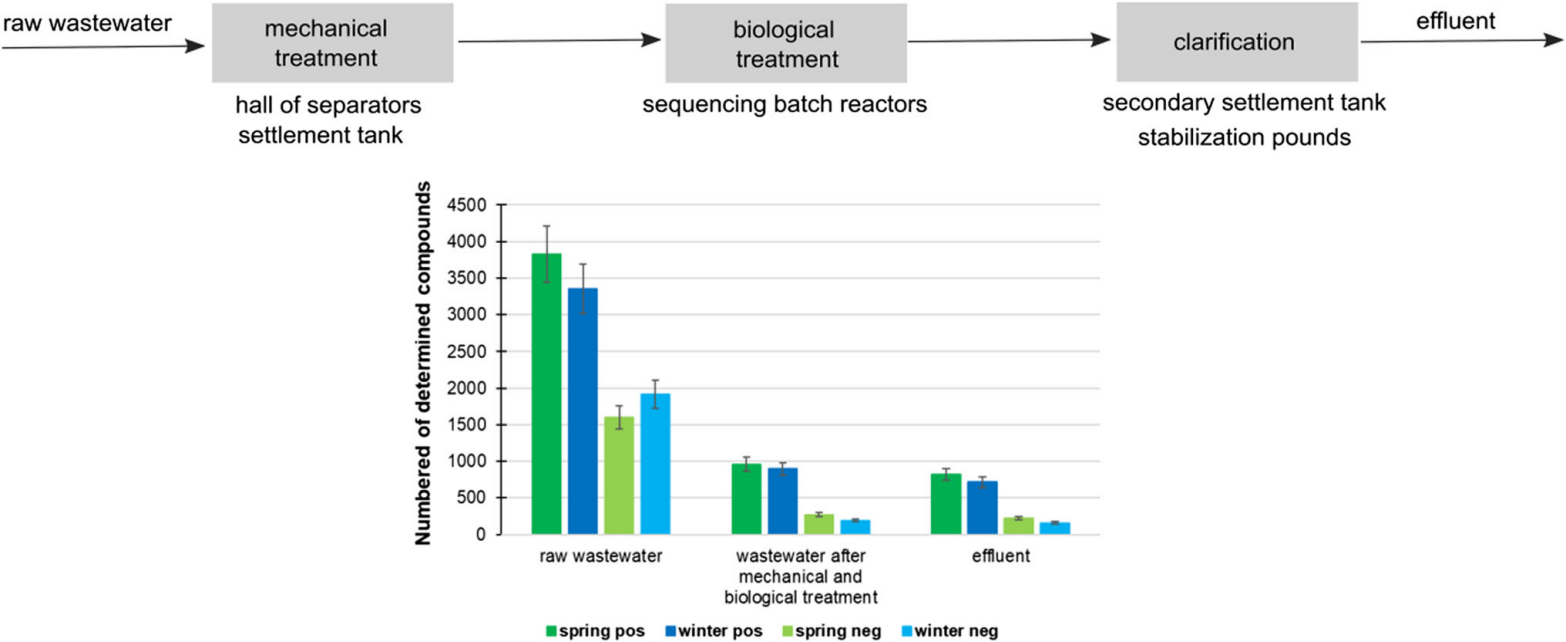
Decline in the content of determined compounds after particular wastewater treatment stage

Decline in the content of determined compounds during different wastewater treatment stage is shown in Fig. 3. Approximately, 80% of the compounds responsible for positive ion formation and 90% of compounds ionizing in negative ion formation mode were removed from raw wastewater as a result of mechanical and biological purification. In samples collected from secondary settlement tank less than 1500 chemical individuals were found. Since then, 15 to 20% of contaminants have been removed from the sewage. However, these samples were collected before the tourists apogee, so that the content of some compounds may fluctuate much. Despite satisfactory results, an improvement of treatment processes is still needed, especially in the context of the use of purified wastewater as a water resource. Among compounds that are not being removed during wastewater treatment processes, there may be some individuals such as emerging contaminants, whose long-term exposure could be harmful to humans. For this reason, the monitoring of these chemicals in wastewater samples is very important.

**Fig. 4:**
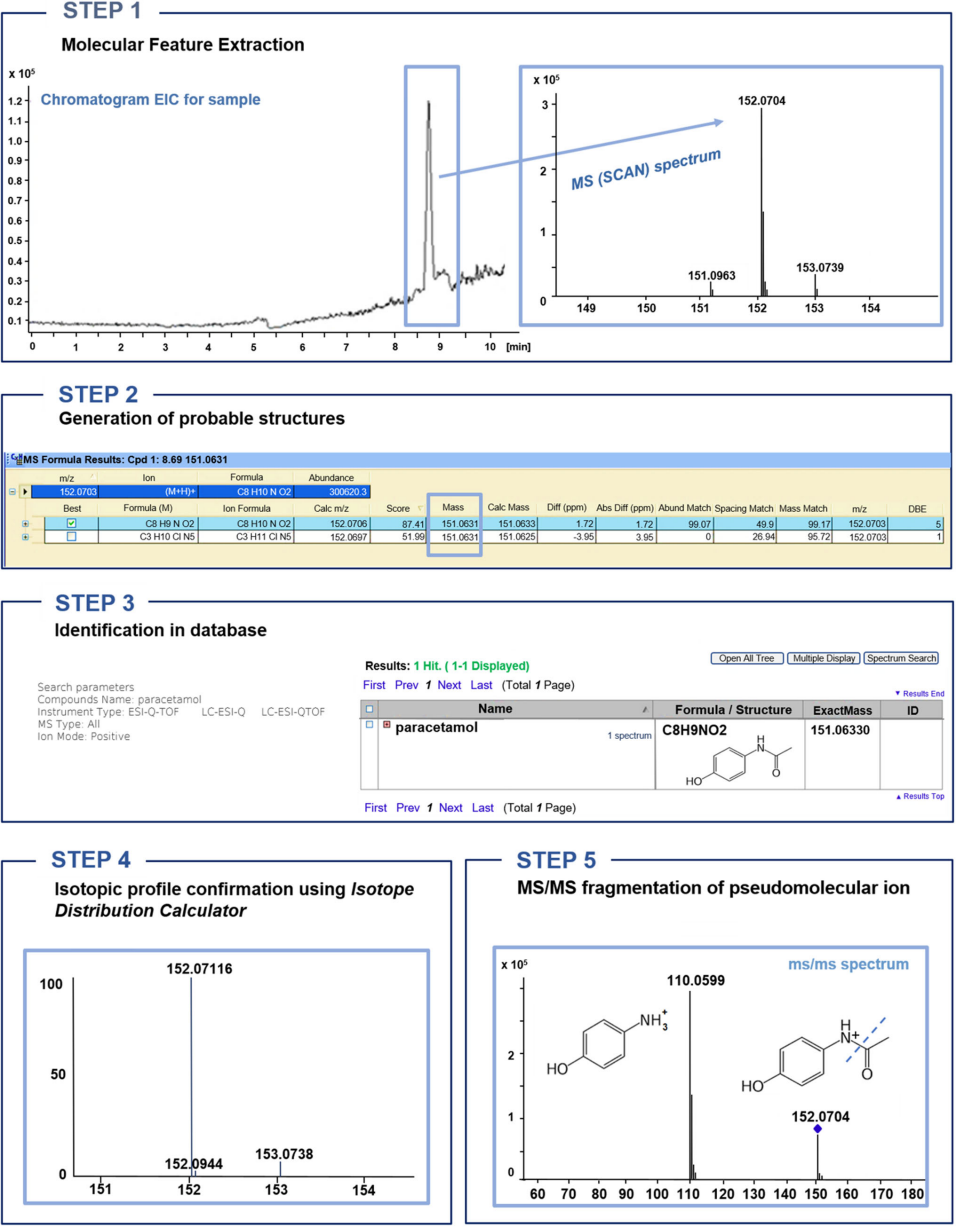
Procedure of the identification of analytes in wastewater

Among all, 12 emerging contaminants that have been commonly detected in wastewater samples all over the world were selected for further consideration. Most of them are commonly used, so their presence in domestic and industrial sewage is worldwide [12–17]. Further investigation has been done to evaluate the presence of selected contaminants in wastewater samples. To isolate them from the wastewater samples, SPE has been performed using three different approaches, dedicated to acidic, neutral, and basic compounds. Therefore, three analytical protocols were applied. They differ in pH of samples and solvent used during elution step. Extracts were analyzed by HPLC–ESI–Q-TOF–MS. Identification of analytes in extracts was based on the procedure presented in Fig. 4. The summary of emerging pollutants detected in wastewater samples identified based on above-mentioned analytical protocol is shown in Table 1.

**Table 1:** The presence of selected contaminants in wastewater samples

Emerging contaminant	Hall of separators			Secondary settlement tank			Effluent quality control station		
	A	B	C	A	B	C	A	B	C
Acesulfame-K	+	+	+	–	+	+	–	+	+
Aspartame	–	–	+	–	–	–	–	–	–
Butyl paraben	–	–	–	–	–	–	–	–	–
Caffeine	+	+	+	–	–	–	–	–	–
Carbamazepine	+	+	+	+	+	+	+	+	+
Cyclamate	+	+	+	–	–	+	–	–	–
Ethyl paraben	+	–	–	–	–	–	–	–	–
Ibuprofen	–	–	+	–	–	–	–	–	–
Methyl paraben	+	–	–	–	–	–	–	–	–
Paracetamol	+	+	+	–	–	–	–	–	–
Saccharin	+	+	+	–	+	+	–	–	–
Sucralose	–	–	+	–	–	+	–	–	+

+, detected; −, not detected

*A* extraction protocol focused on wastewater samples with pH ~ 2, *B* extraction protocol focused on wastewater samples with pH ~ 6, *C* extraction protocol focused on wastewater samples with pH ~ 8

Among the selected pollutants, only butylparaben was not detected at all. According to the decision of the Scientific Committee on Consumer Products (SCCP) of the European Union, the maximum concentration of this compound in consumer products should be lower than 0.19%. Moreover, because of insufficient data to perform risk assessments, the usage of butylparaben in products dedicated to children younger than the age of three is forbidden [18]. It seems that the level of this compound in sewages is probably too low to be detected using HPLC–Q-TOF–MS. Two artificial sweeteners (acesulfame-K and sucralose) and one pharmaceutical residue (carbamazepine) were found in effluent samples. The presence of carbamazepine in all wastewater samples is not extraordinary. This pharmaceutical is often used for treatment of epilepsy, neuropathic pain, schizophrenia, or bipolar disorder and due to growing number of people with mental illnesses it is constantly detected in the sewages [15, 17, 19–21].

Comparing extraction approach C to the two other ones applied for sample preparation step, this approach seems to be the most suitable for the determination of emerging contaminants, in particular artificial sweeteners. In this case, pH of the samples was not corrected (pH ~ 8), while during the elution step three eluents were used. Application of eluent (methanol) additive in the form of ammonia and ethyl acetate together with acetone provided enhanced recovery. The use of SPE-based approach A did not confirm the occurrence of acesulfame-K in wastewater samples from secondary settlement tank and effluent quality control, whereas the use of approach B allowed to detect only six selected compounds in raw wastewater samples. Nevertheless, the application of different SPE cartridges could be considered for the future in order to obtain better recovery of contaminants such as paracetamol, ibuprofen, or parabens.

SPE has been selected for analytes isolation and enrichment from wastewater samples, not only because it requires low sample volumes and a low solvent amount, can be easily automated and no emulsion formation is observed, but also this particular technique represents the most efficient technique to overcome matrix effects (ME %). This is due to the fact that sample pretreatment can be performed with many different sorbent beds or solvents to selectively extract the analytes or elute the impurities; therefore, we have studied three different approaches to find the right combination for efficient extraction, satisfied recovery, and very low ion suppression. A thorough evaluation of matrix effect for wastewater was thus performed by comparing the peak area of the target compound in extract (*A*_matrix_) spiked at 1 μg dm^−3^ (after previous subtraction of the peak area of the analyte presented in the extract) with the peak area of the analyte in the solvent (*A*_solvent_, MeOH/H_2_O 1:1, v/v) at the same concentration level. The percentage of matrix effect was then calculated according to the equation:

$$ {\text{ME}} \% \, = \,(A_{\text{matrix}} /A_{\text{solvent}} \, - \, 1)\, \times \, 100\% . $$

Calculations were performed in triplicate. The results for the different samples indicate that ion suppression was observed for most selected analytes. The lowest suppression was observed for saccharin (− 19.27%), however, for parabens and other artificial sweeteners matrix effects were in the range from − 90 to − 70%. Low ion enhancement was observed for paracetamol (12.47%), whereas in case of caffeine the enhancement of signal was high up to 32%.

Comparing chromatographic signals obtained for wastewater before and after purification, much lower intensity of the peaks has been recorded in case of purified wastewater samples, which may indicate lower concentrations of acesulfame-K, carbamazepine, and sucralose. Probably, these contaminants are not completely removed during wastewater treatment. Due to this fact, they may enter to the environment with effluent discharges to the nearest water basin. Therefore, acesulfame-K, carbamazepine, and sucralose might be selected as markers of anthropogenic pollution of environmental waters in Northern Poland.

## Conclusion

For all these countries that are trying to achieve the balance between economic development and environmental sustainability and protection, wastewater may represent valuable and easily available supply. However, conventional wastewater treatment processes still require improvement. Although over 70% of compounds are being removed during treatment processes, wastewater treatment plants are still not adapted to dispose many contaminants belonging to emerging ones. Lots of them are components of daily use products (e.g., food, beverages, or personal care products), so their concentration in the sewages increases systematically with the number of consumers. Due to their resistance to treatment processes, more and more emerging contaminants are released to the environment with wastewater disposal and entering to reservoirs of drinking water. Their continuous presence in tap water may be harmful, in particular for children. However, the effects of long-term consumption of most of them are still unknown. To determine the emerging contaminants in wastewater samples, three different SPE-based approaches were established and evaluated. Approach C seems to be the most suitable for extraction of these compounds from sewage. The results confirmed the reports that acesulfame-K, carbamazepine, and sucralose are not completely removed during wastewater treatment processes and may be used as markers specific to Polish domestic sewage. Moreover, detection of these contaminants in effluent samples proved that RP-HPLC–Q-TOF–MS can be successfully applied as powerful tool not only for wastewater profiling, but also for the selection and monitoring of pollution markers. Nevertheless, further research on profiling of wastewater and detection of emerging contaminants in treated wastewater is still required.

## Experimental

Acesulfame-K was purchased from Nutrinova (Frankfurt, Germany). Caffeine, paracetamol, carbamazepine, saccharin, ibuprofen, and three parabens (methyl, ethyl, and butyl) were purchased from Sigma-Aldrich (St. Louis, USA). Cyclamate was obtained from Merck (Darmstadt, Germany), sucralose was purchased from Nestlè (Vevey, Switzerland), whereas aspartame was purchased from Ajinomoto (Zug, Switzerland). The internal standard (IS), sodium *N*-(2-methylcyclohexyl)sulfamate, was obtained by synthesis [22] (Department of Organic Chemistry, Faculty of Chemistry, Gdańsk University of Technology). Acetonitrile (HPLC grade), methanol (HPLC grade), and formic acid (> 98%) were purchased from Merck (Darmstadt, Germany). Ethyl acetate (LC–MS grade) and acetone (LC–MS grade) were obtained from Sigma-Aldrich (St. Louis, USA). Methanol (LC–MS grade) and acetonitrile (LC–MS grade) were purchased from VWR Chemicals (Radnor, USA). Ultrapure water was prepared using HPL5 system from Hydrolab (Wiślina, Poland). Ammonium solution (analytical grade) was purchased from Chempur (Piekary Śląskie, Poland) and ethyl acetate (> 99%) was obtained from Sigma Aldrich (St. Louis, USA).

### Sampling

Average daily wastewater samples were collected in February and May 2017 from local wastewater treatment plant located in Northern Poland (Pomeranian Voivodeship). They were collected from three facilities of wastewater treatment plant (WWTP): hall of separators, secondary settlement tank, and effluent quality control station. This wastewater treatment plant is using mechanical, chemical, and activated biological treatment. In the summer season, this WWTP purify about 14,000 m^3^ of sewage per day, whereas after the season almost three times less. This place is surrounded by tourist towns and villages located nearby the Baltic Sea and received generally domestic and industrial discharges, especially from food industry (e.g., fish processing or production of sauces).

## Sample preparation

All collected wastewater samples were kept in glass bottles and stored at 4 °C until the extraction (not longer than 48 h). Three different extraction approaches were evaluated. They were based on the solid-phase extraction (SPE) and performed using Strata- X 33 μm Polymeric RP cartridges from Phenomenex (Torrance, USA). Three different procedures were based on the literature [6, 7, 14]. In whole cases, it was decided to resign from the washing step in order not to lose the analytes. The following procedures were used:

- Approach A:

50 cm^3^ of unfiltered samples (pH ~ 8) were acidified to pH ~ 3 and the 10 mm^3^ of IS was added to the samples. Then, the SPE cartridges were conditioned with 6 cm^3^ of methanol, 3 cm^3^ of ultrapure water, and 3 cm^3^ of acidified ultrapure water (pH ~ 3) prior to use. Wastewater samples were loaded to SPE tubes using special 50 cm^3^ syringes and afterwards, the cartridges were allowed to dry for 15 min under vacuum. Finally, the analytes were eluted gravitationally with 10 cm^3^ of methanol.

- Approach B:

50 cm^3^ of unfiltered samples (pH ~ 8) was acidified to pH ~ 6 and the 10 mm^3^ of IS was added to the samples. Subsequently, the SPE cartridges were conditioned with 6 cm^3^ of methanol, 3 cm^3^ of ultrapure water, and 3 cm^3^ of slightly acidified ultrapure water (pH ~ 6). After loading the samples to SPE tubes, the cartridges were dried for 15 min under vacuum. Then, the analytes were eluted gravitationally with 10 cm^3^ of methanol.

- Approach C:

10 mm^3^ of IS were added to the unfiltered samples (pH ~ 8). Then, the SPE cartridges were conditioned with 5 cm^3^ of methanol and 5 cm^3^ of ultrapure water. After loading the samples, the cartridges were dried for 25 min under vacuum and then, the analytes were eluted with 6 cm^3^ of methanol, 3 cm^3^ of methanol, acetone, and ethyl acetate mixture (2:2:1 v/v/v) and 3 cm^3^ of methanol containing 5% ammonia (all collected to one probe and mixed).

All extracts were collected and evaporated to dryness under the gentle stream of nitrogen. Furtherer, the extracts were reconstructed in 1 cm^3^ of mobile phase and followed by filtration with Puradisc™ 13 mm PTFE (0.2 μm pore size) syringe filters from Sigma-Aldrich (St. Louis, USA). Finally, they were analyzed by HPLC–Q-TOF–MS system.

### Instrumentation

The RP-HPLC–Q-TOF analysis was performed using the Agilent LC system equipped with a binary pump, an online degasser, an autosampler and a thermostated column compartment coupled with the 6450 Q-TOF–MS with Dual ESI in source (Agilent Technologies, Santa Clara, USA). LiChrospher 100 RP-18e (250 × 4.6 mm; 5 μm, Merck, Darmstadt, Germany) column was used in order to separate the analytes. Two different solvent mixtures were examined and applied as a mobile phase: one method has been focused on methanol and water mixture with formic acid (0.1% v/v) and the second one was based on acetonitrile and water (both acidified with formic acid, 0.1% v/v). In both cases, the gradient elution was: 5% of B in 0 min, 0–20 min linear increase from 5 to 100% of B and then 100% of B for 5 min. The last step was conditioning of the column for 5 min. The flow rate of mobile phase was 0.7 cm^3^ min^−1^ and the injection volume was 2 mm^3^. The column temperature throughout the separation process was kept at 40 °C. The ESI source operated with the positive and negative ion mode. The fragmentor voltage was set at 100 V and the mass range was set at 100–1700 in mass spectrometer. The remaining parameters are presented in Table 2. The Q-TOF–MS system was calibrated on a daily basis. In the present study, extracts from the wastewater samples were analyzed using SCAN and targeted MS/MS mode.

**Table 2:** Parameters of HPLC–Q-TOF–MS system

Q-TOF–MS parameters							
Nebulizer gas/psi	Capillary voltage/V		Drying gas flowrate/dm^3^ min^−1^		Temperature of drying gas/°C		
35	3500		10		300		
	Compound	Polarity	Retention time/min		*m/z* of experimental ion	*m/z* of theoretical ion	Error/ppm
			I	II			
Monitored ion and their parameters (scanning mode)							
Artificial sweeteners	Acesulfame	–	5.6	5.5	161.9861	161.9861	0
	Aspartame	–	15.8	19.7	293.1136	293.1137	0.34
	Cyclamate	–	6.3	8.7	178.0535	178.0538	1.68
	Saccharin	–	6.2	7.5	181.9910	181.9912	1.10
	Sucralose	–	8.2	11.8	395.0074	395.0067	− 1.77
Pharmaceuticals	Caffeine	+	7.6	11.2	195.0875	195.0876	0.54
	Carbamazepine	+	12.8	16.9	237.1023	237.1022	− 0.42
	Ibuprofen	–	17.8	20.9	205.1227	205.1229	0.98
	Paracetamol	+	6.8	8.7	152.0704	152.0706	1.32
Parabens	Butyl paraben	–	16.7	19.3	193.0868	193.0870	1.03
	Ethyl paraben	–	13.7	16.5	165.0560	165.0557	− 1.82
	Methyl paraben	–	11.2	14.8	151.0404	151.0401	− 1.99
Mobile phase composition: I-A: H_2_O + 0.1% FA, B: ACN: 0.1% FA; II-A: H_2_O + 0.1% FA, B: MeOH							

	Compound	Polarity	Retention time/min	Ion transition	Collision energy/V
Monitored ion and their parameters (targeted MS/MS mode)					
Artificial sweeteners	Acesulfame	–	5.5	161.98 → 82.03	16.5
	Aspartame	–	19.7	293.11 → 261.08	21.7
	Cyclamate	–	8.7	178.05 → 79.95	17.1
	Saccharin	–	7.5	181.99 → 105.96	17.3
	Sucralose	–	11.8	395.00 → 359.00	15.0
Pharmaceuticals	Caffeine	+	11.2	195.08 → 138.06	17.8
	Carbamazepine	+	16.9	237.10 → 194.10	19.5
	Ibuprofen	–	20.9	205.12 → 160.88	18.2
	Paracetamol	+	8.7	152.07 → 110.06	16.1
Parabens	Butyl paraben	–	19.3	193.08 → 92.03	17.7
	Ethyl paraben	–	16.5	165.05 → 92.03	16.5
	Methyl paraben	–	14.8	151.04 → 92.03	16.0
Mobile phase composition: A: H_2_O + 0.1% FA, B: MeOH					
